# Supplementary material for: Radiofrequency Irradiation Attenuated UVB-Induced Skin Pigmentation by Modulating ATP Release and CD39 Expression
Source: Int J Mol Sci. 2023 Mar 14;24(6):5506. doi: 10.3390/ijms24065506 (PMC10052073; doi:10.3390/ijms24065506)
Supplement: Supplementary file 1 [file ijms-24-05506-s001.zip › ijms-2287374-supplementary.pdf]

International Journal of *Molecular Sciences*

MDPI

Article

Radiofrequency Irradiation Attenuated UVB-Induced Skin Pigmentation by Modulating ATP Release and CD39 Expression

Kyung-A Byun [1,†], Hyoung Moon Kim [2,†], Seyeon Oh [3], Kuk Hui Son [4,*] and Kyunghee Byun [1,3,*]

1 Department of Anatomy & Cell Biology, College of Medicine, Gachon University, Incheon 21936, Republic of Korea
2 Maylin Clinic, Ilsan 10391, Republic of Korea
3 Functional Cellular Networks Laboratory, Lee Gil Ya Cancer and Diabetes Institute, Gachon University of Medicine, Incheon 21999, Republic of Korea
4 Department of Thoracic and Cardiovascular Surgery, Gachon University Gil Medical Center, Gachon University, Incheon 21565, Republic of Korea
* Correspondence: dr632@gilhospital.com (K.H.S.); khbyun1@gachon.ac.kr (K.B.); Tel.: +82-32-460-3666 (K.H.S.); +82-32-899-6511 (K.B.)
† These authors contributed equally to this study.

Citation: Byun, K.-A.; Kim, H.M.; Oh, S.; Son, K.H.; Byun, K. Radiofrequency Irradiation Attenuated UVB-Induced Skin Pigmentation by Modulating ATP Release and CD39 Expression. *Int. J. Mol. Sci.* **2023**, *24*, 5506. https://doi.org/10.3390/ijms24065506

Academic Editor: Andrzej Slominski

Received: 2 March 2023
Revised: 9 March 2023
Accepted: 10 March 2023
Published: 14 March 2023

Copyright: © 2023 by the authors. Licensee MDPI, Basel, Switzerland. This article is an open access article distributed under the terms and conditions of the Creative Commons Attribution (CC BY) license (https://creativecommons.org/licenses/by/4.0/).

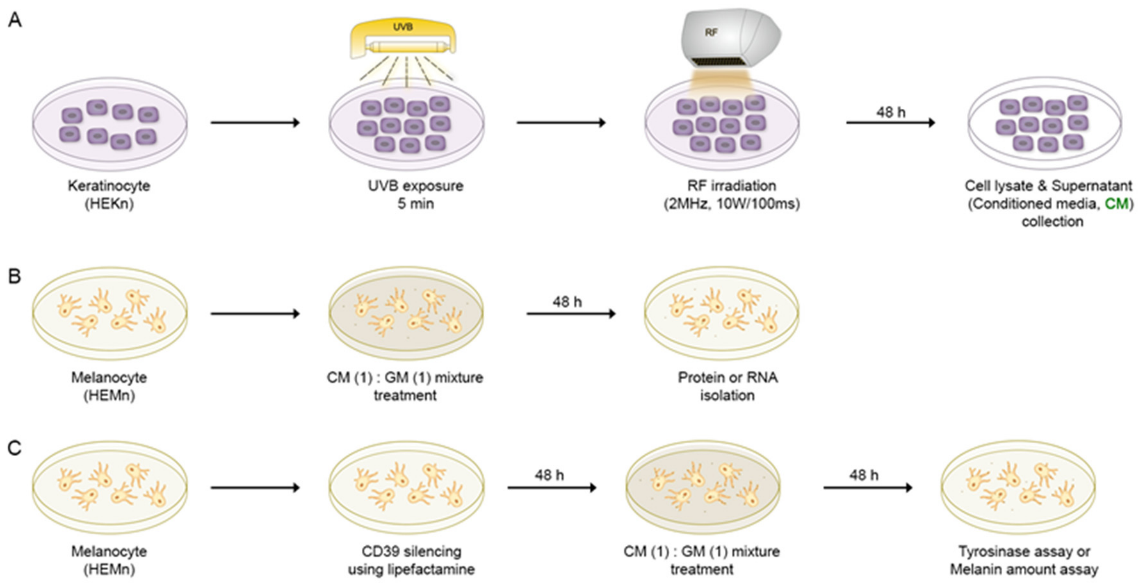

Figure S1. In vitro model schematic summary.

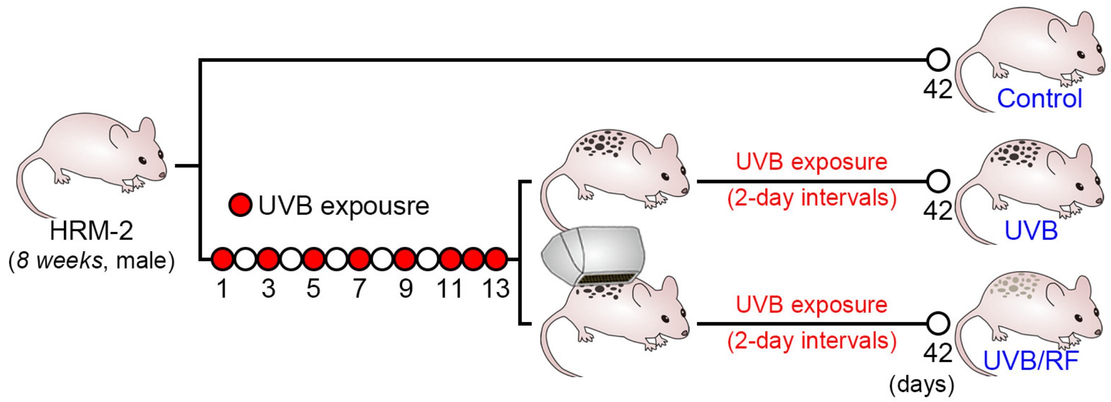

Figure S2. In vivo model schematic summary.

Table S1. List of antibodies for western blotting.

Antibody (host)	Company	Catalog no.	Dilution rate
CD39 (Rabbit)	Proteintech	19229-1-AP	1:4000
CD73 (Rabbit)	Santa cruz	sc-25603	1:2000
$A_{2A}AR$ (Mouse)	Novus bio	NBP1-39474	1:2000
$A_{2B}AR$ (Rabbit)	Prosci	8111	1:500
PKA (Mouse)	R&D system	MAB5908	1:5000
pDRP1 (Rabbit)	BT LAB	BT-AP08624	1:2000
OPA1 (Rabbti)	HUABIO	ET1705-9	1:500
MFN2 (Rabbit)	Abclonal	A12771	1:4000
ERK1/2 (Rabbit)	Cell signaling	9101	1:1000
pERK1/2 (Rabbit)	Cell signaling	9102	1:1000
MITF (Rabbit)	LS Bio	LS-C117668	1:500
pMITF (Rabbit)	BT LAB	BT-AP11347	1:500
β-actin (Rabbit)	Cell signaling	4967	1:1000

Table S2. List of primer for quantitative real time polymerase chain reaction.

Gene		Primers
Actb	Forward	5'-GGACTTCGAGCAAGAGATGG-3′
	Reverse	5'- AGCACTGTGTTGGCGTACAG-3′
PKA	Forward	5'- TCGAACACACCCTGAATGAA -3′
	Reverse	5'- CCAGCGAGTGCAGATACTCA-3′
Creb	Forward	5'- ACTGTAACGGTGCCAACTCC -3′
	Reverse	5'- GAATGGTAGTACCCGGCTGA-3′
